# Monochorionic diamniotic twin brothers with severe hemophilia A: a case report

**DOI:** 10.3389/fped.2026.1770910

**Published:** 2026-02-23

**Authors:** Lorenzo Riboldi, Alessandra Coscia, Chiara Peila

**Affiliations:** Neonatal Unit, Department of Public Health and Pediatric Sciences, University of Turin, Turin, Italy

**Keywords:** case report, congenital hemophilia A, factor VIII, newborn, monochorionic twins

## Abstract

**Background:**

Congenital hemophilia A is a recessive inherited hemorrhagic disorder caused by factor VIII (FVIII) deficiency. According to the activity of functional coagulation FVIII, the severity of hemophilia A is divided into three levels: mild, moderate and severe. The characteristic phenotype in hemophilia is the bleeding tendency. Clinical severity depends on the extent of the FVIII deficiency and the first bleeding episode in severe and moderate congenital hemophilia A usually occurs in early childhood. At present, there are few reports on symptomatic severe congenital hemophilia A in the neonatal period.

**Case presentation:**

We describe a pair of monozygotic twin brothers with severe hemophilia A. Patient-related factors, including a birth weight discrepancy of 10%, the need for non-invasive respiratory support due to mild respiratory distress, duration of breastfeeding, and vaccinations, were similar in both twins. Anti-hemorrhagic prophylaxis was carried out after birth with IM vitamin K. Due to the presence of prolonged bleeding at the sampling site after performing EGA (Blood Gas Analysis) and neonatal screening, a coagulation test was carried out and a coagulation factor assay (dosage of activity of factors VII, IX, XI, XII) was performed accordingly: activated partial thromboplastin time (APTT) was prolonged without extended prothrombin time (PT). Factor VIII activity was completely absent (0.7%) in both twins. Hematological consultancy was requested and a diagnosis of severe congenital hemophilia A was established. Emicizumab was started as the primary anti-hemorrhagic prophylaxis, with good response and no major bleeding events in the first year of therapy.

**Conclusions:**

The coagulation system is not fully developed at birth, complicating clinical decision-making and the correct interpretation of coagulation testing. Targeted coagulation profiling and factor assays are mission-critical for newborns from twin pregnancies when a hematological disorder is suspected. Coagulation factor assays are essential to confirm the diagnosis of hemophilia A. An early diagnosis of hemophilia is crucial for the timely initiation of an appropriate management plan.

## Introduction

Congenital hemophilia A (HA) is an X-linked recessive bleeding disorder caused by factor VIII (FVIII) deficiency. Hemophilia has an overall estimated frequency of approximately one in 10,000 births. HA is more common than hemophilia B (factor IX deficiency), representing 80%–85% of the total hemophilia population, and occurs in 0.01% of newborns (1). The deficiency is the result of mutations in the respective clotting factor gene (F8). The molecular basis underlying HA is well-characterized, and approximately half of severely affected individuals have large genomic inversions disrupting the FVIII gene (F8-Xp28) (2). However, genes are prone to new mutations, and as many as a third of all cases are the result of a spontaneous mutation, with no prior family history (3). Hemophilia generally affects males on the maternal side and is transmitted by heterozygous females, denoted as carriers, who are generally asymptomatic, although cases of symptomatic forms of hemophilia in female carriers have been described (4). Where available and possible, genetic testing for carrier status should be offered to at-risk female family members of people with hemophilia to facilitate genetic counseling and prenatal diagnosis (5). The characteristic phenotype in hemophilia is the tendency to bleed. Clinical severity depends on the extent of the factor VIII deficiency and is inversely related to residual factor VIII activity (FVIII:C) such that patients with less than 1%, 1%–5%, and 5%–30% FVIII:C are classified as severe, moderate, or mild, respectively. While the tendency to bleed is lifelong, children may not have bleeding symptoms until they begin walking or running. In the context of a known family history, patients with HA can often be diagnosed shortly after birth, whereas patients without a positive family history (approximately 30%) are diagnosed significantly later. The median age of the diagnosis of severe HA is 15 months (6), but the median age when the first joint bleed occurs is in the range of 18–20 months (7). In an observational cohort study consisting of 679 patients with severe or moderate HA, the researchers found that the first bleeding episode in those with HA occurred at a median age of 0.82 years in severe disease and 1.47 years in moderate disease (8). However, reports of symptomatic hemophilia in neonatal patients with low FVIII activity are relatively rare.

## Case report

A 39-year-old woman with a monochorionic diamniotic twin pregnancy at 27 weeks and 6 days of gestational age was found to have suspected preterm premature rupture of membranes (pPROM) during a routine prenatal care assessment, for which antibiotic prophylaxis and respiratory distress syndrome (RDS) prophylaxis were performed. An ultrasound assessment of fetal biometry detected intrauterine growth restriction (reduction in abdominal circumference and femoral length) for the first twin, whose umbilical artery Doppler velocimetry was abnormal (pulsatility index >95th centile), with preserved end-diastolic flow. The second twin had normal growth and umbilical artery Doppler velocimetry. No sign of twin-to-twin transfusion syndrome or twin anemia–polycythemia sequence was found.

The combined test was negative. Maternal serologies [HBsAg (Hepatitis B surface Antigen), HCV (Hepatitis C Virus), HIV (Human Immunodeficiency Virus), Toxoplasma, CMV (cytomegalovirus), and LUE/VDRL (Venereal Disease Research Laboratory)] were negative. The Group B Streptococcus (GBS) vagino-rectal swab was negative. The mother was diagnosed with gastroesophageal reflux disease (GERD) during pregnancy and treated with omeprazole.

None of the family members, including the mother, exhibited hemophilia or had abnormal plasma FVIII:C (maternal factor VIII activity was 51.3%) and von Willebrand factor (VWF) values, suggesting no familial inheritance for any hematological disease (in particular, the mother’s International Society for Thrombosis and Hemostasis bleeding assessment tool score was 0).

At 36 weeks and 1 day of gestational age, due to the transverse position of the second twin, a cesarean section was scheduled. The children had a birth weight discrepancy of 10% (First twin: 2,180 g and second twin: 1,950 g). The Apgar score was 9 at the first minute and 9 at the fifth minute for both.

At birth, they both presented mild respiratory distress and, for this reason, respiratory support with CPAP (Fi02 21%) was started and continued for a total of 6/10 h after birth. CPAP support was then suspended with maintenance of stable vital signs on room air. Anti-hemorrhagic prophylaxis was carried out routinely with vitamin K 1 mg IM.

Due to the presence of prolonged bleeding at the sampling site after performing EGA and neonatal screening, in the absence of other specific risk factors in the medical history and clinical course, coagulation tests were carried out and a coagulation factor assay was performed accordingly. The blood test results showed that activated partial thromboplastin time (APTT) was prolonged without extended prothrombin time (PT). Factor VIII activity in both twins was completely absent (FVIII activity: 0.7%) and the activity of factors IX, XI, and XII was low: in the first twin, the activity of factors IX, XI, and XII was 40%, 40%, and 23%, respectively, while in the second twin, this was 36%, 37% and 22%, respectively. Hematological consultancy was requested, and a diagnosis of severe congenital HA was established in both twins. The levels of the other coagulation factors were considered to be within normal limits, given the prematurity of the twins, without indication to repeat the tests.

The Congenital Hemorrhagic Diseases Expert Centre did not provide any immediate therapeutic indications. During their hospital stay, the twins maintained adequate vital parameters without the need for hemodynamic support. They both received enteral nutrition from day 1 of life with maternal/donated milk with regular increases in weight and good food tolerance. They further maintained valid diuresis with a good hydroelectrolyte balance.

The twins presented with a normal objective neurological examination and, on ultrasound brain scans, both presented with mild dysmorphism of the frontal horns and mild dilatation of the lateral ventricles, for which brain MRI was subsequently performed at 40 weeks of gestational age with no hemorrhagic evidence or other significant abnormalities.

They were discharged at 7 days of age (37 + 1 weeks of gestational age) with home therapy comprising vitamin K, multivitamins, and iron supplementation per os.

At 20 days of life, the twins started primary anti-hemorrhagic prophylaxis treatment with emicizumab, a humanized bispecific monoclonal antibody approved for the treatment of hemophilia A, at a standard dosage of 3 mg/kg subcutaneously every 2 weeks.

During hospitalization, NeoGen neonatal screening was performed, and the results were negative. More specifically, regarding hemophilia A, this test includes class 4 and mutations (intronic inversions of 22 and 1; large deletions or nonsense mutations, which cause the total or near absence of the functional protein). Considering their medical history and following the negative NEOGEN result, approximately 11 months after birth, a genetic examination was performed in both twins.

Due to the absence of a family history of hematological disorders and the maternal factor VIII level, a complete genetic sequencing analysis of the F8 gene was proposed not only for both twins but also for their mother in order to exclude her being a healthy carrier of hemophilia A. These gene analyses for mutations in the F8 gene are currently still ongoing.

At follow-up evaluations, they presented with adequate growth and neurodevelopment at 24 months of age. After 2 years of therapy with emicizumab, coagulation assays indicated a normalized PTT ratio of 0.69, their factor VIII activity had increased to 3%, and no factor VIII inhibitors have been detected. Neither of the twins has presented with episodes of spontaneous bleeding.

## Discussion

Congenital hemophilia A is an inherited hemorrhagic disorder caused by a deficiency of the X-linked chromosome factor VIII (FVIII). It occurs in 0.01% of newborns and generally affects males on the maternal side, transmitted by heterozygous females, denoted as carriers, who are generally asymptomatic. DNA-based mutation analysis to identify the specific mutation responsible for hemophilia in a particular family is becoming technically easier and more widely available. This facilitates the identification of carriers and prenatal diagnosis for male fetuses. In the literature, it is reported that 30% of patients with hemophilia A have *de novo* mutations in the FVIII gene (F8) and do not have a family history of hemophilia A (3–11).

Even though the disease is extremely rare in females, a few cases have been documented and are caused by different pathophysiologic mechanisms that are usually genetically based, resulting in genomic disruption in the FVIII gene (12).

For instance, Valleix et al. described a concordant skewing of X-inactivation toward the paternally derived (normal factor VIII gene) chromosome in monozygotic (MZ) female twin pairs with clinical manifestations of severe hemophilia (13). In another study, a pair of MZ twin girls carrying the same novel factor VIII gene mutation had discordant diagnoses of severe and mild hemophilia A, respectively, due to non-random X-chromosome inactivation, where the most severely affected twin exhibited a higher percentage of inactivation of the normal X chromosome (14).

Therefore, after examining the relationship between chorionicity and hemophilia, the available evidence indicates that phenotypic expression is driven by random X-chromosome inactivation and shows no direct correlation with chorionicity itself.

The symptoms of HA are determined by residual FVIII activity in the body. It has been found that the lower the FVIII activity is, the more frequent the bleeding episodes. If the biological activity of factor VIII is less than 1%, the hemophilia is classified as severe and presents with frequent episodes of spontaneous bleeding that occur in approximately 50%–60% of patients (15). These can occur following minor trauma or are secondary to surgery or dental extractions. If the biological activity of factor VIII is between 1% and 5%, the hemophilia is classified as moderate and if the biological activity is between 5% and 40%, the hemophilia is classified as mild (16). The median age of diagnosis of severe HA is 15 months (17). However, reports of neonatal patients with low FVIII activity are relatively rare.

Acute joint hemorrhage is the most common symptom. Intracranial hemorrhage (ICH) is a severe complication of the disease and is the first symptom in 1%–4% of patients. Intracranial bleeds are serious, life-threatening events and are associated with considerable neurological sequelae in survivors (18). In 2021, Zwagemaker et al. conducted a systematic review and meta-analysis summarizing data from 45 studies. They calculated the peak incidences among the neonates and the pooled ICH cumulative incidence was 2.1% (95% CI, 1.5–2.8) per 100 live births. Hence, 2.1% of newborns with hemophilia are expected to experience an ICH in the neonatal period and are 44 times more likely to experience an ICH compared with the general population (19).

It is well-known in the literature that, in monochorionic pregnancies, transfusion imbalances may develop because of the vascular anastomoses that are invariably present. Intertwin transfusion through placental anastomoses causes alterations in cerebral blood flow, leading to an increased risk for brain injuries, such as ICH (20).

Currently, there is no evidence in the literature that a concomitant diagnosis of congenital HA in monochorionic twins influences the occurrence of intracranial bleeds and brain damage in the affected fetuses.

Understanding the clinical features of hemophilia and the appropriateness of the clinical diagnosis and management is essential. According to guidelines published by the World Federation of Hemophilia (WFH) in 2013, a blood test can be used as a first screening test to identify the potential cause of bleeding (i.e., platelet count, bleeding time, platelet function screening tests, PT, and APTT). A confirmation of the diagnosis can be obtained using coagulation factor assays to demonstrate FVIII or FIX deficiency and other appropriate specific investigations, such as informational gene tracking and/or measurement of fetal FVIII:C level (21, 22). Overall, a low FVIII:C in HA is related to race and age and is determined by chromosomal and genetic testing.

The interpretation of coagulation testing in neonates is still challenging. The coagulation system is not fully developed at birth and matures during the first months of infancy, complicating clinical decision-making within hemostasis. Moreover, coagulation parameters at birth differ according to gestational age (GA) (23). To ensure more accurate interpretations of coagulation testing, several studies have been conducted to describe new GA-dependent reference intervals for common coagulation parameters in newborns, as they currently rely on reference intervals defined from cohorts of patients under 1 year of age (24).

In this context, in newborns from twin pregnancies with suspected hematological disorders, a targeted analysis of coagulation parameters and coagulation factor assays are crucial. As for coagulation testing, the results of the coagulation assays are highly age-dependent and must be used to ensure the correct evaluation of bleeding risk in children, especially in the first year of life (25).

Managing patients with HA is complex and the treatment of these patients must involve a comprehensive approach coordinated by a multidisciplinary group of specialists to obtain long-term results (26).

The WFH strongly recommends the use of plasma-derived or recombinant coagulation factor concentrates for the treatment of hemophilia and other inherited bleeding disorders (27).

The standard of care in HA requires regular, prophylactic replacement of factor VIII for bleeding prophylaxis and treating acute bleeding episodes. It is recommended to start as soon as possible, ideally within the first 2 years of life, and before the occurrence of the first joint bleed (28). Due to its short half-life (8–12 h), administration of a standard half-life (SHL) FVIII concentrate is required at least three times per week or every other day to achieve “full prophylaxis.” This is very effective in protecting patients and allowing an almost normal quality of life. However, even if the prophylaxis is started early and provides continued protection against joint damage, it is not sufficient to completely prevent long-term joint disease (29).

The development of neutralizing antibodies (inhibitors) against FVIII is one of the most severe complications in modern severe HA therapy, leading to the FVIII substitution being ineffective and an increased risk of bleeding, mortality, and morbidity. Several genetic and environmental factors have been reported to play a role in inhibitor development and approximately 30% of patients with severe HA develop these anti-factor VIII antibodies (30, 31).

In this context, emicizumab was licensed for bleeding prophylaxis in patients with HA as the first approved non-replacement therapy (NRT). Emicizumab is a bispecific humanized monoclonal antibody, bridging activated FIX and FX and thus mimicking the function of activated FVIII. Due to this mechanism of action, severe HA is reduced to a mild form with an estimated FVIII activity of at least 9% (32).

Emicizumab was created to circumvent the challenges associated with frequent intravenous administration of FVIII and to offer a standardized treatment option for patients with HA. Initially, it was approved by the Food and Drug Administration (FDA) in 2017 for patients with congenital HA in combination with inhibitors and was subsequently approved for patients with HA without factor VIII inhibitors. Its efficacy, safety, and pharmacokinetics were verified in several phase 3 clinical trials, in which it was found to be largely safe and very effective for the prevention of bleeding episodes and reduction in annual bleeding rate (ABR) across the pediatric age spectrum (33–36).

This therapy completely normalizes the PTT, after which it can no longer be used for diagnostic purposes other than to exclude the presence of antibodies (a rare occurrence in <1% of treatments). However, the need remains for on-demand treatment of bleeding episodes with factor VIII. In our case, after 2 years of therapy with emicizumab, coagulation assays indicated a normalized PTT ratio of 0.69, factor VIII activity of 3%, and absent factor VIII inhibitors. Neither of the twins presented with episodes of spontaneous bleeding.

## Conclusion

In conclusion, this article outlines the case of monochorionic diamniotic twin brothers diagnosed at birth with severe congenital HA, which is rarely described in the literature. It is, therefore, important to maintain a high index of suspicion for this condition in neonatal cases of persistent bleeding, even when the family history is unremarkable. The interpretation of coagulation tests in newborns remains inherently complex, as the hemostatic system is developmentally immature and continues to evolve throughout early infancy, thereby complicating clinical decision-making. In this context, targeted coagulation profiling and factor assays are mission-critical for newborns from twin pregnancies when a hematological disorder is suspected.

Early diagnostic assessment is imperative when evaluating neonatal bleeding disorders. Coagulation factor assays are essential to confirm the diagnosis and lead to the timely initiation of an appropriate management plan. Within the neonatal care pathway, clinicians should consistently go beyond standard coagulation screening and request specific factor measurements. Genetic testing should be performed as early as feasible to evaluate the disease risk and inform the clinical strategy. Managing HA is complex and the treatment of patients with hemophilia A must comprise a comprehensive approach and an integrated care model to obtain long-term results. Long-term clinical outcomes depend on a multidisciplinary framework with a strategic focus on neurodevelopment, particularly to identify potential determinants of neurodevelopmental impairment. The continuous monitoring of early developmental milestones is crucial, especially in monochorionic pregnancies, where the intrinsic risk of fetal intracranial hemorrhage is elevated. This further reinforces the strategic need for structured, long-term follow-up as a core pillar of care in monochorionic twin pregnancies.

## Data Availability

The original contributions presented in the study are included in the article/Supplementary Material; further inquiries can be directed to the corresponding author.
